# AI competency misalignment in preventive medicine: a multi-stakeholder survey with latent profile analysis in Sichuan and Chongqing

**DOI:** 10.3389/ijph.2026.1610073

**Published:** 2026-09-10

**Authors:** Qiuyu Pan, Nian Liu

**Affiliations:** 1 School of Public Health, North Sichuan Medical College, Nanchong, China; 2 Department of Radiology, Affiliated Hospital of North Sichuan Medical College, School of Medical Imaging, North Sichuan Medical College, Nanchong, China

**Keywords:** artificial intelligence, latent profile analysis, public health, university-employer collaboration, Western China

## Abstract

**Objective:**

Evaluate supply-demand misalignment in public health artificial intelligence (AI) workforce education in Sichuan and Chongqing, inland Western China.

**Methods:**

Surveyed 1,031 stakeholders (150 employers, 680 students, 201 educators) using Importance-Performance Analysis (IPA) and Latent Profile Analysis (LPA) to quantify skill deficits. Multivariate models assessed collaborative training and faculty transfer factors, guided by a conceptual framework integrating demand, supply, and training perspectives. All coefficients are associational, not causal.

**Results:**

IPA showed employers prioritised risk assessment; students focused on algorithmic construction. LPA on six practical skills identified two profiles: High-Order Application Group (23.2%) and Foundation-Weak Group (76.8%); the weighted combination of profile means reconciled with the overall sample mean. Employers’ deficit perception was positively associated with their collaboration willingness (β = 0.235, 95% CI [0.061, 0.410], p < 0.01). Institutional innovation negatively moderated the link between faculty AI proficiency and research mentorship (β = −0.120, [-0.216, −0.024], p < 0.05).

**Conclusion:**

AI education may overemphasize computational skills relative to frontline operational demands. Mitigation may require stratified pedagogy, real-world data, and less administration. Multi-stakeholder framework is valuable; causality requires further research.

## Introduction

The integration of artificial intelligence (AI) into public health frameworks has progressed from isolated clinical diagnostics to comprehensive, system-level interventions. Recent evidence highlights the utility of machine learning algorithms in addressing complex epidemiological challenges. For instance, interpretable predictive modeling has been utilized to stratify the risk of chronic comorbidities such as obesity and depression [1–3]. Concurrently, digital governance initiatives at the urban level have demonstrated positive associations with medical service performance and preventive health management [4]. Furthermore, predictive analytics leveraging electronic medical records are increasingly deployed to forecast and mitigate treatment interruption among vulnerable populations, including individuals in HIV care [5, 6]. These applications indicate that AI technologies can optimize resource allocation and enhance continuous disease surveillance.

Despite these technological advancements, the rapid diffusion of AI within public health systems introduces substantial implementation risks [7]. The efficacy and safety of AI-driven surveillance and governance frameworks are contingent not merely on algorithmic sophistication, but fundamentally on institutional capacity and workforce readiness [8]. A key challenge in this digital transition is the human capital deficit, characterized by a structural mismatch between the supply of and demand for interdisciplinary professionals possessing concurrent expertise in preventive medicine and AI methodologies [9, 10].

Recent scholarly efforts have sought to define the competencies required for AI-ready public health professionals. Scoping reviews have mapped AI competency development in public health education [11], and frameworks such as the WHO’s Public Health Intelligence Competency Framework [12] and the European digital public health competency framework [13] have delineated core domains including data literacy, analytics/AI, and ethical practice. However, most frameworks remain generic and have not been systematically operationalized in undergraduate preventive medicine education, particularly in non-Western contexts. Furthermore, empirical evidence on interdisciplinary curriculum evaluation and workforce readiness assessment in resource-constrained settings remains scarce [14]; these competencies are grounded in established digital literacy frameworks [15, 16].

Current investments predominantly target digital infrastructure, whereas the educational ecosystem for public health practitioners has not evolved proportionally [17]. Market-demanded competencies—such as epidemic prediction and algorithmic fairness—are frequently marginalized in conventional public health curricula [18]. This educational discrepancy may present a risk: the deployment of advanced computational models by an inadequately trained workforce could lead to analytical misinterpretations, exacerbate existing health disparities, and compromise data security protocols.

Consequently, identifying and mitigating the structural factors contributing to this talent mismatch is imperative for the sustainable implementation of AI in population health. Using a cross-sectional design within Sichuan Province and Chongqing Municipality—representative regions of Western China—this study investigates the supply-demand dynamics of AI competencies in the public health sector. By applying Importance-Performance Analysis (IPA) to quantify specific skill deficits and Latent Profile Analysis (LPA) to delineate student competency profiles, this research identifies key features of the current educational misalignment.

**FIGURE 1:**
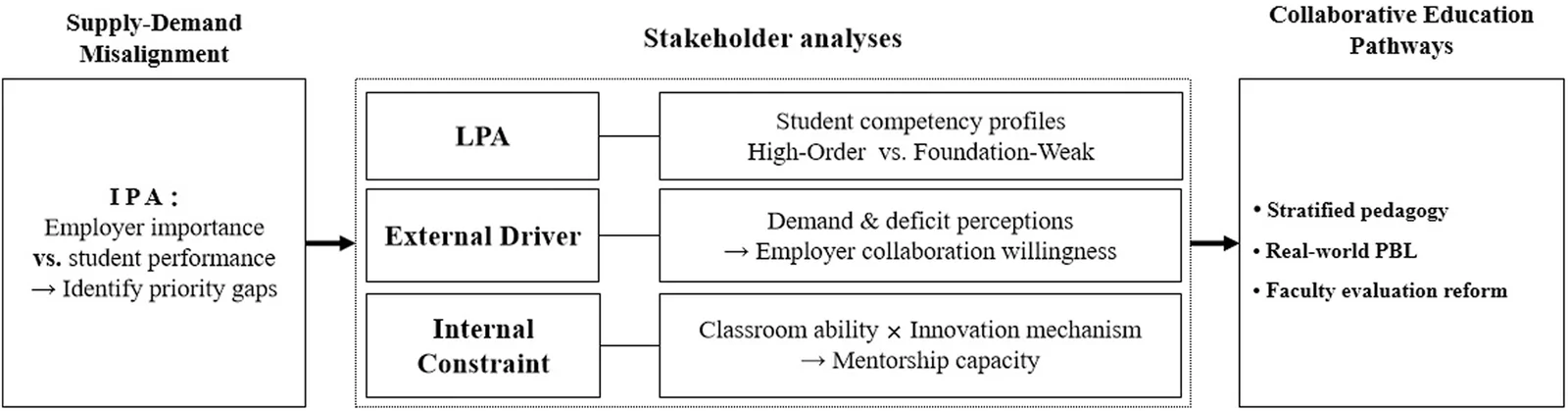
Conceptual framework for multi-stakeholder analysis of AI workforce education (Sichuan and Chongqing, China, 2026).

A conceptual framework (Figure 1) was developed to guide the analysis, integrating demand identification (IPA), supply diagnosis (LPA), and training optimization (regression/moderation). This framework integrates employer, student, and educator perspectives. The findings may inform a cross-sectoral collaborative training framework, aligning academic curricula with practical public health demands and thereby contributing to a workforce foundation that may help address the risks of AI implementation.

## Methods

### Study design and participants

A cross-sectional survey was conducted between March and April 2026 in Sichuan Province and Chongqing Municipality, in accordance with the Declaration of Helsinki and approved by the Institutional Ethics Committee (NSMC Ethics Project Review [2025] No.040). The study population comprised three cohorts: employer (demand side), undergraduate preventive medicine and public health (supply side), and faculty/administrators (training side). Data were collected digitally via the Wenjuanxing platform (https://www.wjx.cn) from 7 universities and 141 employer organizations (including CDCs, hospitals, health centers, government departments, research institutes, and medical enterprises).

After obtaining electronic informed consent from all participants, eligible respondents met predefined inclusion criteria (belonging to one of the three target populations and able to complete the survey). Exclusion criteria included implausibly short completion times (<120 s), logical contradictions, and patterned responses. Of 1,058 initial responses, 27 were excluded, yielding a final effective sample of 1,031 participants (150 employers, 680 students, 201 educators), with an effective response rate of 97.4%.

### Measures

Based on literature review and expert consultation, initial item pools were generated and refined through two rounds of pilot testing (cognitive interviews, n = 11; small-sample pilot, n = 45). Items with low relevance, unclear wording, or substantial overlap were deleted, resulting in final instruments: Employer (25 items), Student (20 items), and Teacher (22 items), all using a 5-point Likert scale.

The Employer Demand Scale (25 items, total α = 0.920) covered five dimensions: Talent Demand Intensity, Current Status Assessment, Perceived Competency Deficits, Training Expectations, and Industry-Education Cooperation Willingness.

The Student Self-Assessment Scale (20 items, total α = 0.901) covered five dimensions: Cognition/Attitude, Basic Knowledge, Practical Application Skills, Innovation/Collaboration, and Continuous Learning.

The Teacher Competency Scale (22 items, total α = 0.874) covered five dimensions: Curriculum System, Teacher Competency/Innovation Mechanisms, Teaching Resources, Student Ability Evaluation, and Future Strengthening Directions.

Confirmatory Factor Analysis confirmed good psychometric properties, with standardized factor loadings ranging from 0.647 to 0.860, McDonald’s ω from 0.778 to 0.901, and corrected item-total correlations >0.30.

### Statistical analysis

All analyses were performed using R version 4.4.0, with a two-sided significance level of 0.05.

#### Descriptive statistics and importance-performance analysis

Normality was checked and confirmed for all continuous variables. Importance-Performance Analysis (IPA) was employed to construct a two-dimensional matrix [19], with the vertical axis representing employers’ perceived importance and the horizontal axis students’ self-evaluated performance; empirical grand means served as the cross-origin thresholds. Bootstrap resampling and sensitivity analyses using alternative thresholds confirmed the stability of the core findings. Indicators in the upper-left quadrant (high importance, low performance) were defined as high-priority zones for curriculum intervention.

#### Latent profile analysis

Latent Profile Analysis (LPA) using maximum likelihood estimation was conducted on the standardized z-scores of the six practical application skill items (C1–C6), with local independence assumed (EEI parameterization) [20]. Models specifying one to four profiles were evaluated. Model selection was guided by the Bayesian Information Criterion (BIC), sample-size adjusted BIC (aBIC), and Entropy (>0.80), supplemented by the Bootstrap Likelihood-Ratio Test (BLRT) [21, 22]. Sensitivity analysis using an alternative variance-covariance specification (VVI) confirmed identical class assignments, supporting the robustness of the selected solution. All analyses were performed using the tidyLPA and mclust packages in R.

#### Regression and moderation diagnostics

Multiple linear regression and moderation models were constructed to examine factors associated with supply-demand dynamics. For the employer model, the dependent variable was collaboration willingness (E1–E4); independent variables were demand intensity (A1–A5) and perceived deficits (C1–C5). For the teacher moderation model, the dependent variable was research mentorship ability (B5); the independent variable was classroom AI application ability (B4); and the moderator was institutional innovation mechanism (B2). All continuous predictors were grand-mean centered prior to analysis [23], and covariates (organization type, size, AI adoption stage, respondent role for employers; academic rank, teaching years, prior AI training for teachers) were included to adjust for potential confounders.

Regression diagnostics confirmed no multicollinearity (VIF <5), acceptable homoscedasticity, and no influential outliers (Cook’s distance <1). Significant interactions were probed using simple slope analysis.

## Results

### Baseline characteristics and structural misalignment

Supplementary File 1 presents the baseline demographic and professional characteristics of the 1,031 stakeholders included in the final analysis.

On the demand side, the 150 employer representatives were drawn from a broad spectrum of public health sectors, notably hospitals (19.3%), CDCs (16.0%), and research institutes (16.0%). Most employer respondents served as technical or operations heads (64.7%) at municipal (38.7%) or district/county (22.0%) levels. Furthermore, a significant proportion of these organizations had already initiated AI technology integration, either at the initial trial (44.0%) or partial implementation (32.7%) stages.

Among the 680 students, the gender distribution was balanced (51.8% male, 48.2% female). Participants spanned various academic stages, with a robust representation across core public health disciplines, including occupational and environmental health (24.3%), epidemiology and biostatistics (20.7%), and nutrition and food hygiene (20.4%). Notably, a substantial majority of students (75.7%) reported having prior AI-related training or coursework.

For the training supply side, the educator cohort (n = 201) was predominantly composed of academic staff with 89.6% holding either a master’s or doctoral degree. The respondents represented diverse academic ranks and teaching experiences, and 85.1% indicated prior involvement in AI-related teaching or curriculum delivery.

### Descriptive results and perceived competency alignment

Initial descriptive statistics indicated that overall evaluations across the three cohorts—employers, students, and educators—were moderately high. Normality was confirmed for all continuous variables (|skewness| < 2, |kurtosis| < 7). Specifically, employers reported the highest scores regarding the “perceived competency deficits” of current students (3.75 ± 0.88). In contrast, students self-evaluated their “practical application skills” most favorably (3.73 ± 0.97), whereas educators expressed the highest confidence in the current “teaching resource supply” (3.88 ± 0.85).

**FIGURE 2:**
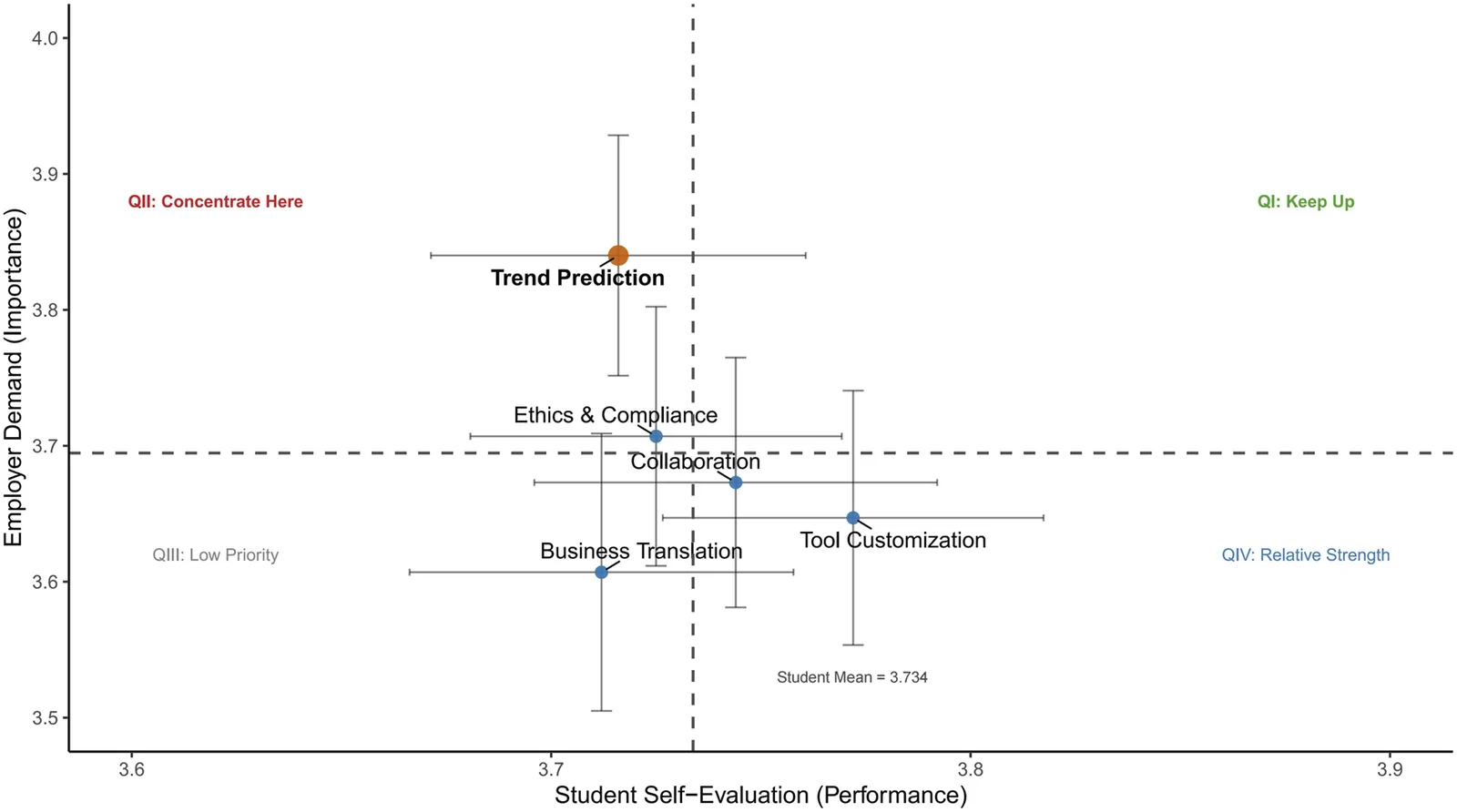
Importance-Performance Analysis of Perceived Competency Alignment (Sichuan and Chongqing, China, 2026). Note: The crosshair lines represent the empirical grand means of student self-evaluation (horizontal) and employer importance ratings (vertical). Quadrants are labeled as follows: QI (Keep Up), QII (Concentrate Here), QIII (Low Priority), QIV (Relative Strength). Error bars indicate 95% bootstrap confidence intervals.

To pinpoint the structural loci of these educational discrepancies, an Importance-Performance Analysis (IPA) matrix was constructed, with empirical grand means established as the cross-origin thresholds following standard IPA practice (Figure 2). Robustness was assessed via bootstrap resampling and sensitivity analyses using alternative thresholds, which confirmed the stability of the core directional pattern. As detailed in Table 1, the data revealed perceptual misalignments between employer priorities and student self-assessed competencies. Most critically, “epidemic trend prediction and risk assessment” emerged as the highest-rated employer priority (3.84 ± 1.06), yet students’ self-reported performance in this domain was comparatively lower (3.72 ± 1.17), placing this competency in the high-priority “Concentrate Here” quadrant (Quadrant II).

**TABLE 1:** Extended Importance-Performance Analysis (IPA) summary with 95% CI and Cohen’s d (Sichuan and Chongqing, China, 2026).

Core competency	Employer item (demand)	Student item (self-evaluation)	Employer mean (95% CI)	Student mean (95% CI)	Difference (95% CI)	Cohen’s d	IPA quadrant
Business understanding & problem translation	A1	C1	3.61 [3.40, 3.80]	3.71 [3.62, 3.80]	−0.11 [−0.31, 0.09]	−0.090	QIII
Tool customization & model construction	A2	C2	3.65 [3.47, 3.83]	3.77 [3.69, 3.86]	−0.13 [−0.34, 0.09]	−0.104	QIV
Trend prediction	A3	C3	3.84 [3.66, 4.01]	3.72 [3.63, 3.80]	0.12 [−0.06, 0.32]	0.108	QII
Ethics, data security & compliance	A4	C4	3.71 [3.52, 3.90]	3.73 [3.64, 3.81]	−0.02 [−0.21, 0.18]	−0.016	QII
Interdisciplinary collaboration & communication	A5	C5	3.67 [3.50, 3.85]	3.74 [3.65, 3.84]	−0.07 [−0.29, 0.13]	−0.058	QIV

Conversely, while students rated their “tool customization and model construction” capacity relatively highly (3.77 ± 1.22), direct demand for this specific technical skill from employers was comparatively moderate (3.65 ± 1.14), mapping it to Quadrant IV. All employer-student differences were small in magnitude; however, bootstrap analysis indicated that the directional pattern—particularly the higher priority assigned to epidemic prediction by employers—remained stable, supporting interpretation of these findings as relative priority differences rather than absolute gaps.

### Unobserved heterogeneity in student skill acquisition

Given the aggregate performance deficits identified in the IPA, an individual-centered Latent Profile Analysis (LPA) was subsequently conducted to decompose the unobserved heterogeneity within students’ practical AI application skills (Items C1–C6). After systematically comparing the goodness-of-fit indices for models specifying one to four latent classes, the 2-class model was identified as the optimal parsimonious solution (Supplementary File 2). This configuration demonstrated the lowest Bayesian Information Criterion (8679.390) and sample-size adjusted BIC (8619.063), alongside robust classification accuracy (Entropy = 0.999).

The 2-class model was robust to alternative specifications, including ordinal latent class analysis and VVI covariance, and outperformed a one-factor CFA model (ΔBIC = 2971.7), supporting the categorical distinction over a single continuous trait.

**FIGURE 3:**
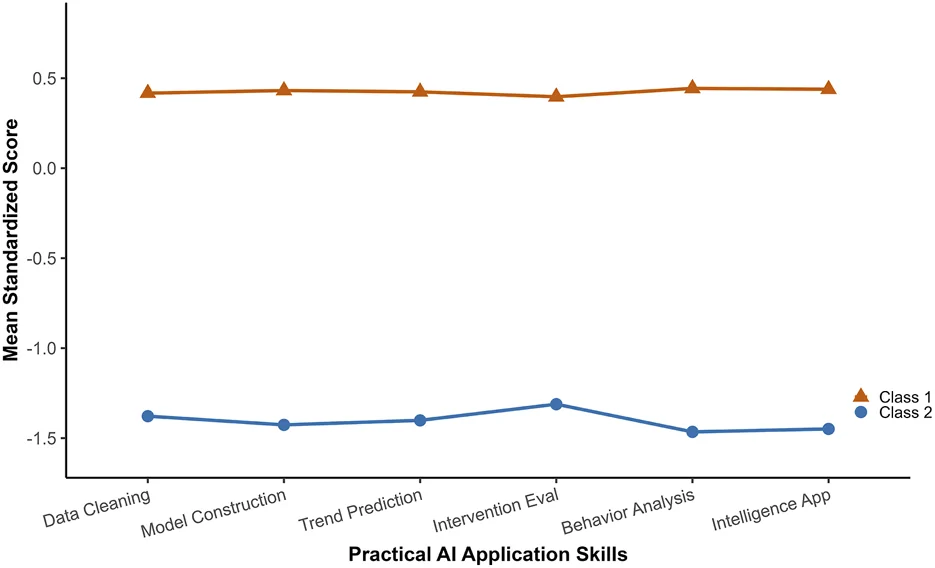
Latent Profile Plot of Students’ Practical AI Application Skills (Sichuan and Chongqing, China, 2026). Note: Solid lines represent the mean standardized scores for each of the two identified profiles: Class 1 (High-Order Application, n = 158, 23.2%) and Class 2 (Foundation-Weak, n = 522, 76.8%). The y-axis indicates mean standardized scores.

Based on the conditional mean distributions extracted from the optimal model (Figure 3), the digital skill proficiency among the student cohort exhibited distinct polarization, yielding two distinct subpopulations. The *High-Order Application Group* (Class 1, n = 158, 23.2%) maintained consistently high performance across all six skill indicators (mean range: 4.18–4.30), demonstrating comprehensive readiness to integrate AI into public health scenarios. Conversely, the *Foundation-Weak Group* (Class 2, n = 522, 76.8%) scored substantially lower across all dimensions (mean range: 1.96–2.21). This indicates that the digital literacy of the vast majority of current public health students appears limited, lacking the substantive technological capacity required by hiring organizations.

### Factors associated with university-employer collaboration

To evaluate how the aforementioned competency deficits influence external stakeholders, a multiple linear regression model was formulated to ascertain factors associated with employers’ willingness to participate in collaborative academic-practice training. The overall model yielded a statistically significant fit (F = 18.89, p < 0.001, Adjusted R^2^ = 0.194) (Table 2).

**TABLE 2:** Multiple linear regression predicting employers’ willingness for university-employer cooperation (Sichuan and Chongqing, China, 2026).

Variables	B	Se	β	t	p	95% CI	VIF
(Intercept)	1.638	0.349	—	4.688	<0.001	[0.948, 2.329]	—
AI talent demand intensity (X1)	0.327	0.088	0.308	3.725	<0.001	[0.154, 0.501]	1.259
Perceived student competency deficits (X2)	0.235	0.088	0.220	2.667	0.009	[0.061, 0.410]	1.259

Model Fit: R^2^ = 0.204, Adjusted R^2^ *= 0.194, F = 18.89, p* < 0.001. *n = 150.*

To account for potential confounders, the employer regression model was expanded to include organization type, organization size, AI adoption stage, and respondent role as covariates. After controlling for these variables, Demand_X1 (B = 0.363, p < 0.001) and Deficit_X2 (B = 0.211, p = 0.023) remained significant positive predictors of cooperation willingness, confirming the robustness of the core findings. Among covariates, only government departments showed significantly lower willingness compared to CDC (B = −0.589, p = 0.027). Model R^2^ = 0.303 (Adjusted R^2^ = 0.219).

The regression parameters indicated that demand-side perceptions of educational inadequacies were positively associated with cooperation willingness. Specifically, both a heightened “talent demand intensity” (β = 0.327, p < 0.001) and a higher perception of current students’ “competency deficits” (β = 0.235, p = 0.009) served as significant positive predictors. These findings suggest that overarching talent scarcity and the explicit observation of skill mismatches are associated with greater willingness to proactively intervene in the university pedagogical process.

### Internal moderation effects in faculty capacity transfer

Internally, the successful reform of AI curricula relies heavily on faculty capabilities. A moderation model was subsequently constructed to test the boundary conditions governing the translation of educators’ “personal classroom AI application ability” into their “ability to mentor student research projects,” utilizing “institutional innovation mechanisms” as the moderating variable (Supplementary File 3).

To address potential confounding in the teacher model, academic rank, years of teaching, and prior AI training were added as covariates. The interaction term (classroom ability × innovation mechanism) remained significant after adjustment (B = −0.125, p = 0.013), consistent with the unadjusted model. All covariates were non-significant (p > 0.05). Model R^2^ = 0.418 (Adjusted R^2^ = 0.388).

Regression diagnostics for the teacher moderation model showed all VIF values below 2, indicating no multicollinearity. The Breusch-Pagan test was significant (p < 0.001), suggesting mild heteroscedasticity; however, ordinary least squares estimates are robust to moderate violations. All Cook’s distance values were below 1 (maximum = 0.175), confirming no influential outliers. Residual analysis supported the overall model assumptions. No severe violations of regression assumptions were detected.

**FIGURE 4:**
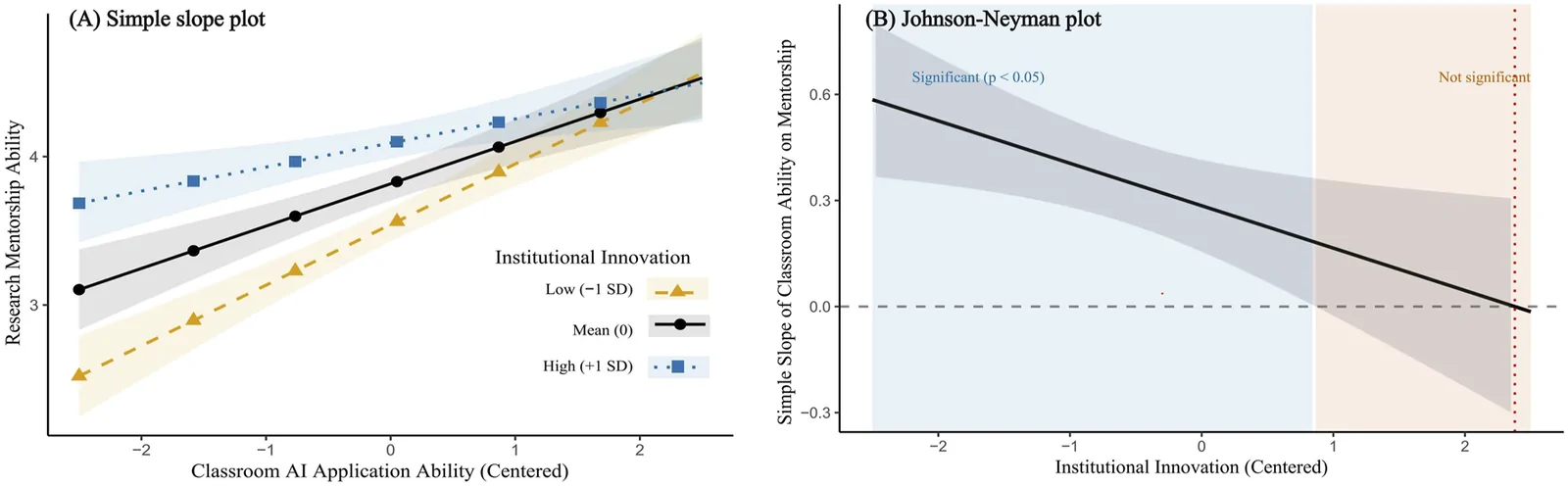
Moderation Effect of Institutional Innovation Support on Faculty Capacity Transfer (Sichuan and Chongqing, Western China, 2026). Note: Panel **(A)** presents the simple slope plot showing the conditional association between classroom AI application ability (centered) and research mentorship ability at low (−1 SD), mean, and high (+1 SD) levels of institutional innovation support. Shaded areas represent 95% confidence bands. The positive association was significant at low (slope = 0.408, p < 0.001) and mean (slope = 0.285, p < 0.001) levels, but non-significant at high levels (slope = 0.162, p = 0.101). Panel **(B)** displays the Johnson-Neyman plot showing the simple slope across the full range of the moderator (centered). The shaded regions indicate where the slope is statistically significant (p < 0.05). The transition point (W = 2.378) falls outside the observed moderator range (−2.826 to 1.174). All variables were measured on 5-point Likert scales and centered prior to analysis. n = 201.

While the main effect remained significantly positive, the core interaction term (Classroom Capacity × Innovation Mechanism) yielded a significantly negative coefficient (β = −0.120, t = −2.468, p = 0.014). To elucidate the trajectory of this negative moderation, a simple slope analysis was executed (Figure 4A). Simple slope analysis revealed that the positive association between classroom AI application ability and research mentorship was significant at low (slope = 0.408, p < 0.001) and mean (slope = 0.285, p < 0.001) levels of institutional innovation, but became non-significant at high levels (slope = 0.162, p = 0.101). Johnson-Neyman analysis further indicated that the simple slope remained significant across the entire observed moderator range, as the transition point (W = 2.378) fell outside the observed range (−2.826 to 1.174) (Figure 4B). Ceiling effects were ruled out as an alternative explanation, as only 32.1% of teachers in the high innovation group scored the maximum on research mentorship ability (mean = 4.21, SD = 0.62).

The visualization revealed a diminishing association: when institutional innovation support were relatively weak (Mean - 1 SD), educators’ personal classroom capabilities efficiently translated into research mentorship abilities (indicated by a steep, positive slope). Conversely, under high levels of institutional innovation support (Mean +1 SD), the translation efficiency flattened significantly.

## Discussion

This study addresses a critical gap in empirical evidence on AI workforce readiness in resource-constrained educational contexts by systematically assessing multi-stakeholder perceptions of competency alignment. Utilizing empirical multi-stakeholder data from Sichuan and Chongqing, this study examined the structural dilemmas inherent in AI workforce education within preventive medicine and public health. The analyses were guided by a conceptual framework (Figure 1) integrating three stakeholder perspectives: demand identification (employer priorities), supply diagnosis (student competency profiles), and training optimization (educator capacity and institutional support). The findings suggest a notable structural misalignment between the current educational ecosystem of universities and the pragmatic operational demands of frontline employers. During this transition of the public health system towards digitalization—as emphasized by the World Health Organization’s digital health strategy [24]—this misalignment may be rooted in the delayed adaptation nature of existing educational evaluation systems [25].

Examining the demand side, employers’ requirements for public health AI talent have converged on higher-order competencies such as epidemic trend prediction and risk assessment. It was indicated by our regression analysis that the more strongly employers perceive the “competency deficits” of graduates, the greater their willingness to participate in joint university-employer training. Frontline operational departments express a need for interdisciplinary professionals capable of transforming multi-source health data into actionable intelligence [26, 27]. The findings on workforce readiness align with recent European evidence showing that AI training for the public health workforce remains dominated by short-format initiatives rather than comprehensive competency-based programs [28]. The polarized student profiles we observed—76.8% in the foundation-weak group—suggest that generic competency frameworks may require contextual adaptation for implementation in educational settings [13]. This pragmatic necessity may be associated with collaborative university-employer education, resonating strongly with previous findings on academic-practice integration [29].

The IPA matrix identified potential deviations in curriculum prioritization between the demand and supply sides. The prediction and risk assessment capacity required by employers fell into the high-priority Quadrant II. Conversely, the model construction and tool customization capacity, where students demonstrated high self-efficacy, mapped onto a zone of relative strength. This perceived misalignment suggests a possible overemphasis on algorithmic tool manipulation at the expense of epidemiological critical thinking. However, the effect sizes for all employer-student differences were negligible (Cohen’s d < 0.2), and bootstrap stability was only moderate. Therefore, these findings should be interpreted as directional indications of relative priority rather than definitive evidence of a substantial mismatch.

As perspectives in clinical informatics argue, a key challenge to integrating AI in health systems is not only the mechanical execution of generic algorithms, but also the need for comprehensive understanding into authentic operational scenarios [30–32]. The public health system faces a shortage of interdisciplinary experts who can translate complex domain knowledge into computable tasks while maintaining ethical oversight [11]. Consequently, university AI curricula may need to undergo a shift, moving from a curriculum focused primarily on computational techniques to one centered on public health problem-solving [33]. Quantitative data from this study further suggest that technical instruction without a strong epidemiological foundation may be insufficient to address the competency gaps required for intelligent public health transformation [34].

Furthermore, LPA revealed a notable “digital divide” internal to the supply side. The student cohort exhibited high polarization: only 23.2% attained the high-order application group, while 76.8% were classified into the foundation-weak group. This heterogeneity within the student supply side suggests that uniform pedagogical approaches may be insufficient to address diverse competency levels. One possible interpretation, informed by prior literature [35–37], is that perceived difficulty with computational sciences could contribute to this polarization. However, since AI-related anxiety, prior training, and curriculum content were not directly measured, this remains speculative. Alternative explanations—including self-assessment bias, scale-use differences, unmeasured prior training, institution-level variation, ceiling effects, and common-method variance—should also be considered. Future research could incorporate validated measures and objective assessments to clarify these relationships.

Investigating faculty dynamics revealed a nuanced interaction: institutional innovation support negatively moderated the positive association between classroom AI application ability and research mentorship. The simple slope was significant at low and mean levels of innovation but became non-significant at high levels. Johnson-Neyman analysis confirmed that the association remained significant across the entire observed range of the moderator. Rather than acting as a barrier, high levels of innovation frameworks were associated with attenuating the efficiency with which teachers translate classroom AI application ability into extracurricular research mentorship. Viewed through the Technological Pedagogical Content Knowledge (TPACK) framework [38] and the Task Crowding-out Effect theory [39], one possible interpretation involves competing demands. When administrative incentive structures heavily emphasize novel pedagogical formats, they may divert a portion of faculty time and cognitive resources away from the intensive, individualized task of student mentorship [40]. However, this crowding-out interpretation remains speculative and warrants future research with direct measures of faculty workload and time allocation. Consequently, while institutional innovation initiatives remain essential, a heavy reliance on metrics-driven assessments may be associated with the cross-domain transfer of faculty expertise.

While the present study offers a multi-stakeholder assessment of AI competency alignment, its cross-sectional design and reliance on aggregated institutional data limit the ability to examine how specific university-employer partnerships influence competency development over time. Future research could adopt longitudinal designs that track students across their training and early career stages, linking university curricula, employer demands, and objectively measured competency outcomes. Matched designs that connect specific universities, employers, and students would further clarify the mechanisms through which collaboration and institutional support translate into workforce readiness.

Based on this systemic analysis, three intervention strategies are proposed. First, implement a “precision-stratified” pedagogical system. For the foundation-weak majority, curricula could prioritize AI data ethics and epidemiological logic to reduce anxiety; for the high-order cohort, advanced practical training could be implemented via interdisciplinary platforms [41]. Second, comprehensively deepen interdisciplinary Project-Based Learning (PBL) driven by real-world data. Universities may consider partnering with CDCs to integrate de-identified authentic epidemiological datasets, shifting student evaluation anchors from algorithmic accuracy to the efficacy of resolving actual public health problems [42]. Third, refine the faculty evaluation ecosystem to reduce excessive administrative burdens. Universities may need to systemically refine administrative evaluations, reducing rigid pedagogical metrics [43].

### Study limitations

This study acknowledges certain limitations. Constrained by the cross-sectional design, strict causal inferences cannot be established, despite the deployment of regression and moderation models. Second, the assessment of student skills and faculty capabilities relied primarily on self-reported scales, which may be susceptible to social desirability bias. Third, the analyses were based on aggregated data from multiple institutions rather than matched university-employer-student dyads. Future research may benefit from designs that link specific universities, employers, curricula, and students, or from longitudinal designs that examine whether collaboration and institutional support predict objectively measured competency gains.

### Conclusion

Based on empirical evidence from Sichuan and Chongqing, Western China, this study suggests that the sustainable integration of AI into public health may be associated with a structural misalignment between academic training emphasizing computational techniques and frontline demands centered on public health problem-solving. Furthermore, LPA identified polarization, with 76.8% of students in the Foundation-Weak group and 23.2% in the High-Order Application group, suggesting a need for precision-stratified pedagogy. Rigidly structured administrative frameworks may be associated with lower educators’ effectiveness in translating their AI proficiency into practical research guidance. To address these competency gaps in these regions, universities may consider implementing real-world data-driven project-based learning and further refine faculty evaluation metrics. This multi-stakeholder assessment, guided by the conceptual framework, highlights the value of integrating demand, supply, and training perspectives in examining AI workforce education. Given the cross-sectional nature of this study, all associations reported should be interpreted as correlational, not causal. Further research in other geographic and institutional contexts is needed to assess the generalizability of these findings.

## Data Availability

The datasets generated and/or analyzed during the current study are available from the corresponding author on reasonable request.
