# Supplementary material for: AI competency misalignment in preventive medicine: a multi-stakeholder survey with latent profile analysis in Sichuan and Chongqing
Source: Int J Public Health. 2026 Sep 10;71:1610073. doi: 10.3389/ijph.2026.1610073 (PMC13601018; doi:10.3389/ijph.2026.1610073)
Supplement: Supplementary file 1 [file Supplementaryfile1.docx]

**Supplementary File 1. Baseline Characteristics of the Study Participants (N = 1,031)**

| **Characteristics** | **Category** | **n (%)** |
| --- | --- | --- |
| **1. Students (n = 680)** |  |  |
| **Sex** | Male | 352 (51.8%) |
|  | Female | 328 (48.2%) |
| **Ethnicity** | Han | 383 (56.3%) |
|  | Ethnic Minorities | 297 (43.7%) |
| **Discipline / Major** | Epidemiology and Biostatistics | 141 (20.7%) |
|  | Occupational and Environmental Health | 165 (24.3%) |
|  | Nutrition and Food Hygiene | 139 (20.4%) |
|  | Maternal, Child and Adolescent Health | 133 (19.6%) |
|  | Health Care Management | 102 (15.0%) |
| **Prior AI-related Training** | Yes | 515 (75.7%) |
|  | No | 165 (24.3%) |
| **2. Educators (n = 201)** |  |  |
| **Sex** | Male | 104 (51.7%) |
|  | Female | 97 (48.3%) |
| **Age (years)** | ≤ 30 | 31 (15.4%) |
|  | 31–35 | 65 (32.3%) |
|  | 36–40 | 46 (22.9%) |
|  | 41–45 | 23 (11.4%) |
|  | 46–50 | 16 (8.0%) |
|  | 51–55 | 10 (5.0%) |
|  | > 55 | 10 (5.0%) |
| **Highest Educational Level** | Bachelor's degree or below | 21 (10.5%) |
|  | Master's degree | 82 (40.8%) |
|  | Doctoral degree | 98 (48.8%) |
| **Professional Title** | Junior (e.g., Teaching Assistant) | 83 (41.3%) |
|  | Intermediate (e.g., Lecturer) | 76 (37.8%) |
|  | Associate Senior (e.g., Assoc. Professor) | 28 (13.9%) |
|  | Senior (e.g., Professor) | 14 (7.0%) |
| **Years of Teaching Experience** | < 5 | 42 (20.9%) |
|  | 5–10 | 45 (22.4%) |
|  | 11–20 | 67 (33.3%) |
|  | > 20 | 47 (23.4%) |
| **Prior AI Teaching Experience** | Yes | 171 (85.1%) |
|  | No | 30 (14.9%) |
| **3. Employers (n = 150)** |  |  |
| **Employer Sector** | Center for Disease Control and Prevention | 24 (16.0%) |
|  | Hospital / Maternal and Child Health | 29 (19.3%) |
|  | Community / Township Health Center | 28 (18.7%) |
|  | University / Research Institute | 24 (16.0%) |
|  | Medical / Health Technology Enterprise | 27 (18.0%) |
|  | Government Administrative Department | 18 (12.0%) |
| **Organization Level** | National level | 11 (7.3%) |
|  | Provincial level | 16 (10.7%) |
|  | Municipal level | 58 (38.7%) |
|  | District / County level | 33 (22.0%) |
|  | Township / Community level | 22 (14.7%) |
|  | Other | 10 (6.7%) |
| **Professional Role** | Senior Management / Organization Head | 13 (8.7%) |
|  | Middle Management / Department Head | 15 (10.0%) |
|  | Human Resources / Recruitment Manager | 25 (16.7%) |
|  | Technical / Operations Head | 97 (64.7%) |
| **Organization Size (Employees)** | < 50 | 50 (33.3%) |
|  | 50–200 | 62 (41.3%) |
|  | 201–500 | 24 (16.0%) |
|  | > 500 | 14 (9.3%) |
| **Current Use of AI Technologies** | Not yet implemented | 18 (12.0%) |
|  | Initial trial stage | 66 (44.0%) |
|  | Partial implementation stage | 49 (32.7%) |
|  | Extensive implementation stage | 17 (11.3%) |
