# Supplementary material for: AI competency misalignment in preventive medicine: a multi-stakeholder survey with latent profile analysis in Sichuan and Chongqing
Source: Int J Public Health. 2026 Sep 10;71:1610073. doi: 10.3389/ijph.2026.1610073 (PMC13601018; doi:10.3389/ijph.2026.1610073)
Supplement: Supplementary file 2 [file Supplementaryfile2.docx]

**Supplementary File 2. Fit Indices for Latent Profile Models of Students' Practical AI Application Skills**

| **Number of Profiles** | **Log-Likelihood** | **AIC** | **BIC** | **saBIC** | **Entropy** | **BLRT p** | **Parameter Count** | **Class Sizes** | **Avg. Posterior Probabilities** |
| --- | --- | --- | --- | --- | --- | --- | --- | --- | --- |
| 1 Class | -5786.267 | 11596.534 | 11650.799 | 11612.698 | 1 | — | 6 | 680 | — |
| **2 Classes** | **-4277.735** | **8593.47** | **8679.39** | **8619.063** | **0.999** | **<0.001** | **13** | **522 / 158** | **0.999 / 0.998** |
| 3 Classes | -4273.512 | 8599.025 | 8716.599 | 8634.046 | 0.743 | 0.312 | 20 | 422 / 158 / 100 | 0.832 / 0.799 / 0.841 |
| 4 Classes | -4276.204 | 8618.409 | 8767.638 | 8662.859 | 0.528 | 0.451 | 27 | 159 / 210 / 158 / 153 | 0.731 / 0.675 / 0.702 / 0.715 |
| *Note: Bold row indicates selected optimal model. LPA was performed on standardized z-scores (C1–C6). AIC = Akaike Information Criterion; BIC = Bayesian Information Criterion; saBIC = sample-size adjusted BIC; BLRT = bootstrap likelihood-ratio test. Parameter Count = k × (p + 1) − 1, where k = number of profiles and p = number of indicators (6). Class sizes are shown as n per class; posterior probabilities are shown per class. LMR test was not available in the tidyLPA output; BLRT p-values are reported instead. The 2-class model was selected based on lowest BIC (8679.390), highest Entropy (0.999), and significant BLRT (p < 0.001). n = 680.* | | | | | | | | | |
