# Supplementary material for: AI competency misalignment in preventive medicine: a multi-stakeholder survey with latent profile analysis in Sichuan and Chongqing
Source: Int J Public Health. 2026 Sep 10;71:1610073. doi: 10.3389/ijph.2026.1610073 (PMC13601018; doi:10.3389/ijph.2026.1610073)
Supplement: Supplementary file 3 [file Supplementaryfile3.docx]

Supplementary File 3. Moderation Effect of Institutional Innovation Mechanisms on Faculty Capacity Translation

| **Variables** | **B** | **SE** | **β** | **t** | **p** | **95% CI** | **VIF** |
| --- | --- | --- | --- | --- | --- | --- | --- |
| Intercept | 3.817 | 0.065 | - | 58.683 | < 0.001 | [3.688, 3.945] | - |
| Classroom AI application ability (X) | 0.285 | 0.066 | 0.273 | 4.311 | < 0.001 | [0.155, 0.416] | 1.723 |
| Institutional innovation mechanism (W) | 0.268 | 0.072 | 0.259 | 3.741 | < 0.001 | [0.127, 0.409] | 1.628 |
| **Interaction Term (X × W)** | **-0.120** | **0.049** | **-0.172** | **-2.468** | **0.014** | [-0.216, -0.024] | 1.816 |
| *Note: R² = 0.408, Adjusted R^2^ = 0.399, F = 45.28, p < 0.001. Continuous predictors were mean-centered prior to analysis.n = 201.* | | | | | | | |
